# A large-scale neural network model of the influence of neuromodulatory levels on working memory and behavior

**DOI:** 10.3389/fncom.2013.00133

**Published:** 2013-10-03

**Authors:** Michael C. Avery, Nikil Dutt, Jeffrey L. Krichmar

**Affiliations:** ^1^Department of Cognitive Sciences, University of California IrvineIrvine, CA, USA; ^2^Department of Computer Sciences, University of California IrvineIrvine, CA, USA

**Keywords:** spiking neural networks, working memory, neuromodulation, dopamine, noradrenaline

## Abstract

The dorsolateral prefrontal cortex (dlPFC), which is regarded as the primary site for visuospatial working memory in the brain, is significantly modulated by dopamine (DA) and norepinephrine (NE). DA and NE originate in the ventral tegmental area (VTA) and locus coeruleus (LC), respectively, and have been shown to have an “inverted-U” dose-response profile in dlPFC, where the level of arousal and decision-making performance is a function of DA and NE concentrations. Moreover, there appears to be a sweet spot, in terms of the level of DA and NE activation, which allows for optimal working memory and behavioral performance. When either DA or NE is too high, input to the PFC is essentially blocked. When either DA or NE is too low, PFC network dynamics become noisy and activity levels diminish. Mechanisms for how this is occurring have been suggested, however, they have not been tested in a large-scale model with neurobiologically plausible network dynamics. Also, DA and NE levels have not been simultaneously manipulated experimentally, which is not realistic *in vivo* due to strong bi-directional connections between the VTA and LC. To address these issues, we built a spiking neural network model that includes D1, α2A, and α1 receptors. The model was able to match the inverted-U profiles that have been shown experimentally for differing levels of DA and NE. Furthermore, we were able to make predictions about what working memory and behavioral deficits may occur during simultaneous manipulation of DA and NE outside of their optimal levels. Specifically, when DA levels were low and NE levels were high, cues could not be held in working memory due to increased noise. On the other hand, when DA levels were high and NE levels were low, incorrect decisions were made due to weak overall network activity. We also show that lateral inhibition in working memory may play a more important role in increasing signal-to-noise ratio than increasing recurrent excitatory input.

## Introduction

The prefrontal cortex (PFC) is associated with executive functions and plays a primary role in flexibly controlling activity in other brain regions (Miller and Cohen, 2001). To achieve this in a task-dependent manner, one of the key functions of the PFC is to hold on to information in the absence of sensory stimulation (Goldman-Rakic, 1995). This act of holding onto information is known as working memory and is important for many cognitive processes, including attention and goal-directed actions. The contents of working memory are actively shaped by neuromodulatory systems, including the dopaminergic, noradrenergic, cholinergic, and serotonergic systems, which are highly interactive and ubiquitous in the PFC (Briand et al., 2007). These systems have been shown to be important for online adaptation of behavior in order to guide attention to important stimuli (Krichmar, 2008; Avery et al., 2012). In fact, dopaminergic and noradrenergic depletion in the dlPFC has been shown to be as detrimental as completely lesioning the dlPFC itself (Brozoski et al., 1979). For the purpose of this paper, we will focus on the dopaminergic (DA) and noradrenergic (NE) interactions with the dorsolateral prefrontal cortex (dlPFC), which is a subset of neurons near the principal sulcus in the PFC that are involved in visuospatial working memory.

The DA and NE systems, which originate in the ventral tegmental area (VTA) and locus coeruleus (LC), respectively, have an activity-dependent effect on the dlPFC (Arnsten, 2011). At optimal levels of DA and NE, precise representations of information are held in working memory. As DA and NE levels decrease due to relaxation, fatigue, or sleep, the PFC has less precise and noisier representations of information (e.g., during relaxation) or becomes non-functional (e.g., during sleep). As DA and NE levels increase due to stress (e.g., fight or flight response mode), input to working memory neurons is markedly decreased, making it functionally disconnected from the rest of the brain. These changes in the functionality of the dlPFC due to variations of DA and NE concentrations produce the characteristic “inverted-U” dose-response. That is, working memory is impaired at very high or low concentrations of DA and NE leading to deficits in behavioral performance in terms of the accuracy with which saccades were made (Vijayraghavan et al., 2007; Wang et al., 2007). The DA and NE's ability to rapidly and flexibly adapt synaptic efficacies without changing the network architecture has been suggested to be a new form of plasticity, dubbed Dynamic Network Connectivity, or DNC (Arnsten et al., 2010).

The underlying mechanisms that give rise to DA and NE related changes in working memory are well established (Arnsten et al., 2010), and include: suppression of lateral excitation (D1 dopamine receptors), enhancement of recurrent excitation (α2 adrenergic receptors), and reduction in the overall input to a neuron (D1 and α1 adrenergic receptors). These mechanisms, however, have not been tested and verified in a neurobiologically plausible, large scale network model. Furthermore, experiments typically involve independent manipulation of DA or NE. This disjoint change in neuromodulatory levels is unlikely *in vivo* due to reciprocal connections between the VTA and LC (Sara, 2009).

To verify the current mechanistic understanding of how DA and NE affect working memory, as well as explore changes to working memory and behavior when DA and NE concentrations are both at non-optimal levels, we developed a spiking neural network model of the dlPFC that included simulated D1 dopamine receptors, as well as α2A and α1 noradrenergic receptors. By manipulating the levels of dopamine and noradrenaline in our model, we were able to reproduce the inverted-U profiles seen in experimental studies. We also were able to make predictions of how working memory would change in situations that involve simultaneous manipulations of DA and NE outside of optimal levels. That is, we examined how working memory was altered when DA and NE concentrations were both low, both high, and when one concentration was high and the other was low. Finally, we show how these changes in neuromodulatory levels lead to behavioral deficits. This study helps to verify current circuit-level theories on how D1, α2A, and α1 receptors sculpt PFC activity and provides further predictions of how working memory will change with neuromodulatory levels that have previously been unexplored experimentally.

## Methods

**Figure 1:**
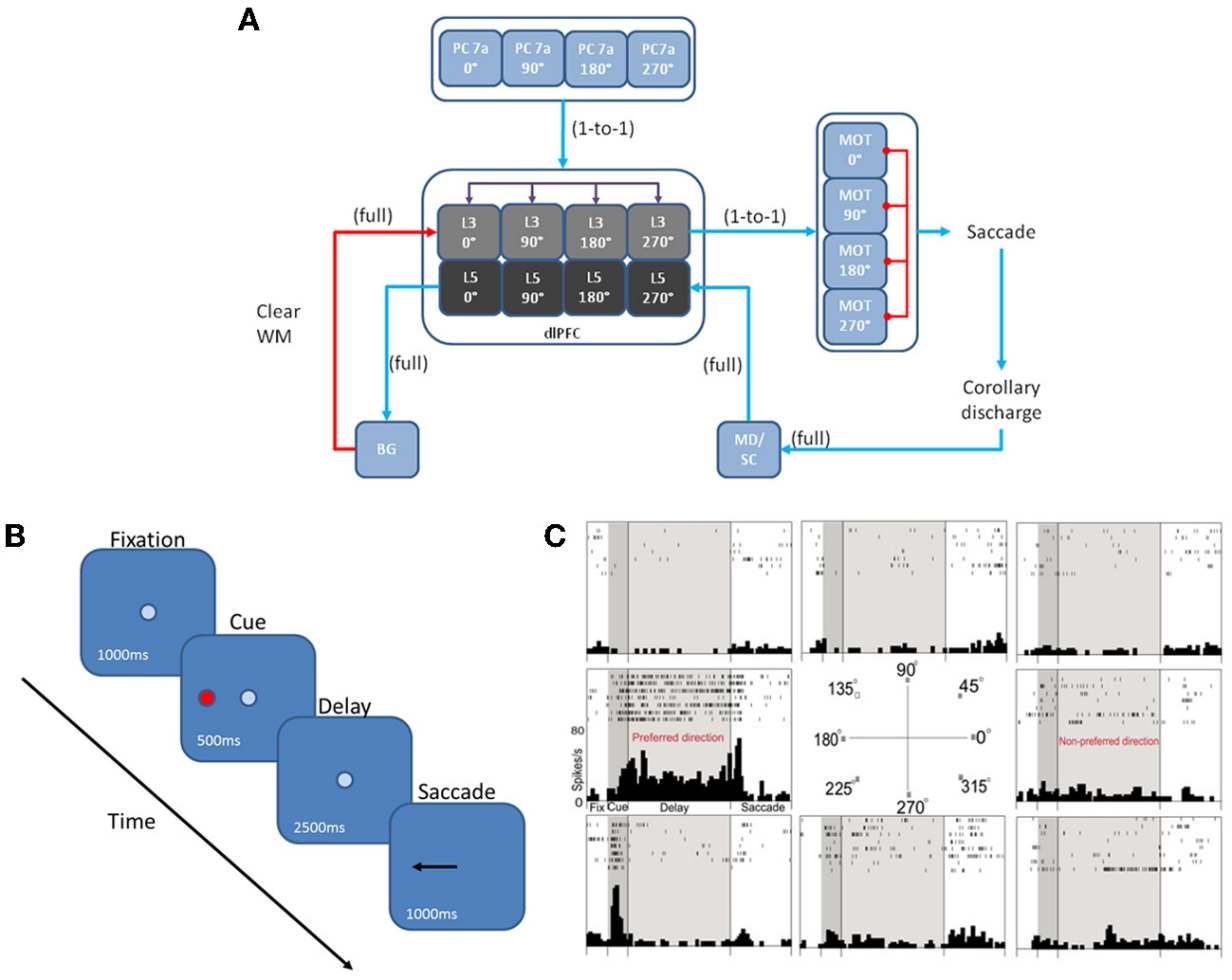
**Network architecture, experiment, and neural responses**. **(A)** The model contained 4 input areas (PC 7a) that projected topographically to layer 3 of four cortical columns (that is, PC neurons coding for 180° projected to layer 3 neurons coding for 180°). The layer 3 neurons also outputted topographically to motor output areas in order to bias motor responses. Layer 5 neurons in each cortical layer received input from the MD/SC in a non-topographic manner. These neurons, in turn, projected to a basal ganglia layer in order to clear working memory after a behavioral response was made. **(B)** We modeled our experiment after the oculomotor delayed response (ODR) behavioral paradigm. This task is broken down into four stages: fixation, cue, delay, and response. The subject must fixate on a visual screen until a cue is briefly presented. After the cue is flashed there is a delay period (2.5 s in our model) during which the subject must remember where the cue was. Lastly, the subject must saccade to the place on the screen where the subject thought the cue was presented. **(C)** Typical response of a recorded neuron in the ODR task. As you can see, the neuron in this case shows persistent activity when a cue is presented at 180°. This is considered the neurons “preferred direction.” This neuron is non-responsive to cues at other spatial locations (non-preferred directions) [adapted from Wang et al. (2007)].

We developed a spiking neural network model that included a dlPFC with four-two layer columns each with a preferred saccade direction, a parietal cortex, basal ganglia, superior colliculus, and four motor output areas (Figure 1A). In addition, the model incorporated dopaminergic and noradrenergic neuromodulation, including simulated D1, α2A, and α1 receptors. We tested our model on the oculomotor delay response (ODR) task, in which a subject must remember the location of a briefly flashed cue over a delay period of 2.5 s then saccade to that location (Figure 1B). Figure 1C shows the firing rate of a PFC neuron that was recorded during an ODR task (Wang et al., 2007). The neuron showed persistent firing during the delay period when the cue was presented at 180°. This is considered the “preferred direction” for this neuron. If the cue was presented at any other spatial location (non-preferred direction), the neuron would not show persistent firing during the delay. This suggests that neural ensembles, of which this neuron is a part of, are holding onto stimulus information in working memory. Our goal was to develop a model that was able to successfully replicate the inverted-U dose-response seen when varying dopamine and norepinephrine levels in ODR tasks order to better understand how neuromodulation affects PFC activity and influences behavior.

### Network model

**Figure 2:**
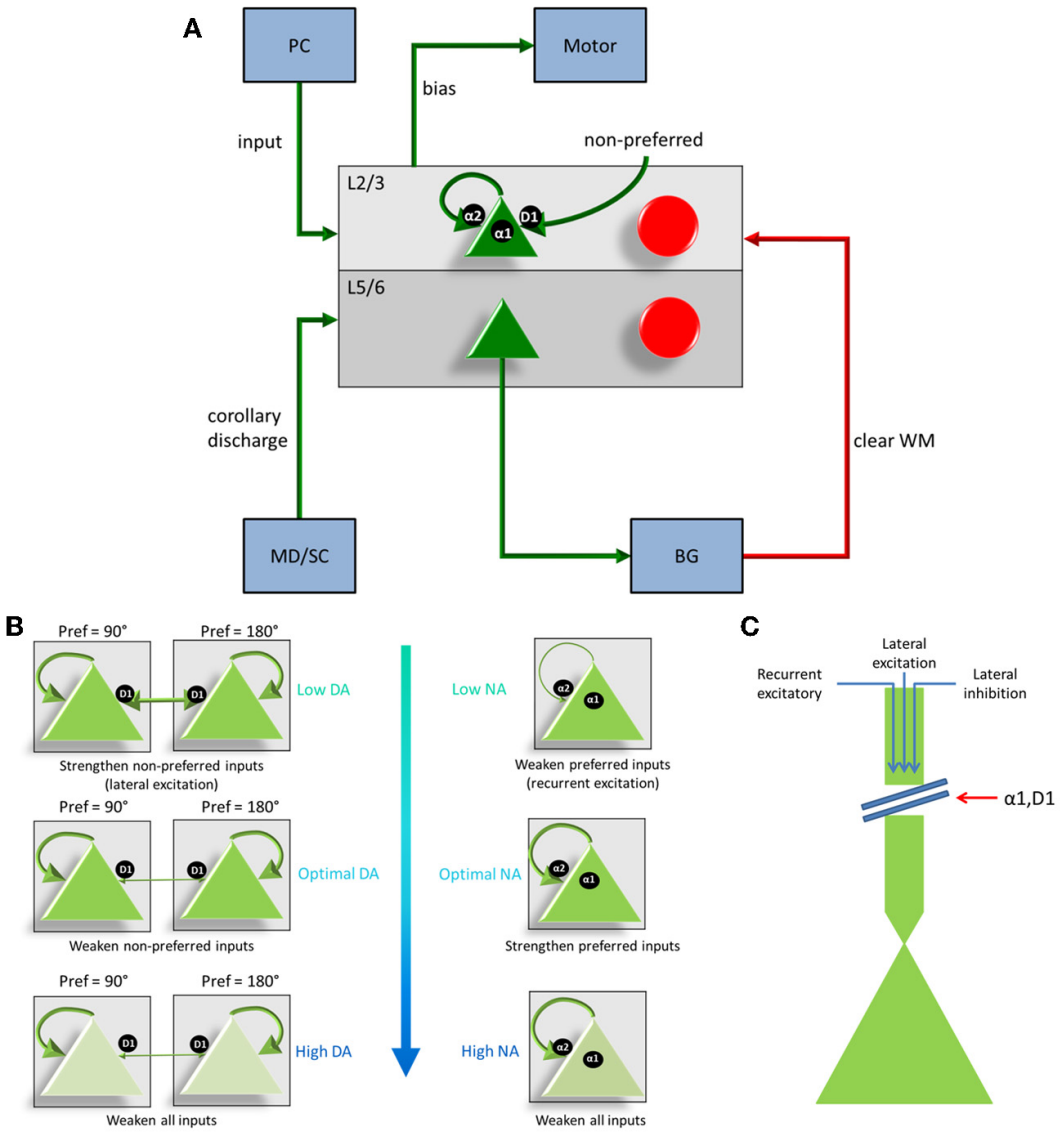
**Individual column architecture and neuromodulatory effects**. **(A)** Within a column in the PFC, neuromodulators were modeled by changing the strength of recurrent excitatory inputs (α2A receptors), inputs from non-preferred directions (D1 receptors), and the overall inputs to the neurons (D1 and α1 receptors) depending on concentrations of dopamine and norepinephrine. As in Figure 1, this architecture also shows how layer 5 neurons in each column received input from the MD/SC and output to the basal ganglia in order to clear working memory. **(B)** On the left and right we show the affects that dopamine and norepinephrine levels have on layer 3 neurons in the columns of our model. When DA is low (top left), connections between columns (non-preferred excitatory inputs) are enhanced, which leads to degradation in spatial tuning. When NE is low (top, right) recurrent excitatory connections are weakened leading to weak firing rates. At optimal levels of DA and NE, non-preferred inputs are blocked from other columns and recurrent excitatory inputs within a column are enhanced. This enhances spatial tuning with the working memory circuits. When DA or NE are high, D1 receptors or α1, respectively weaken all inputs to neurons in layer 3 of the cortical columns. **(C)** Figure demonstrating, in detail, how activation of α1 or overactivation of D1 receptors can block all inputs to layer 3 neurons, including recurrent excitatory inputs within a column, lateral excitatory inputs from other columns, and lateral inhibitory inputs from other columns.

**Figure 3:**
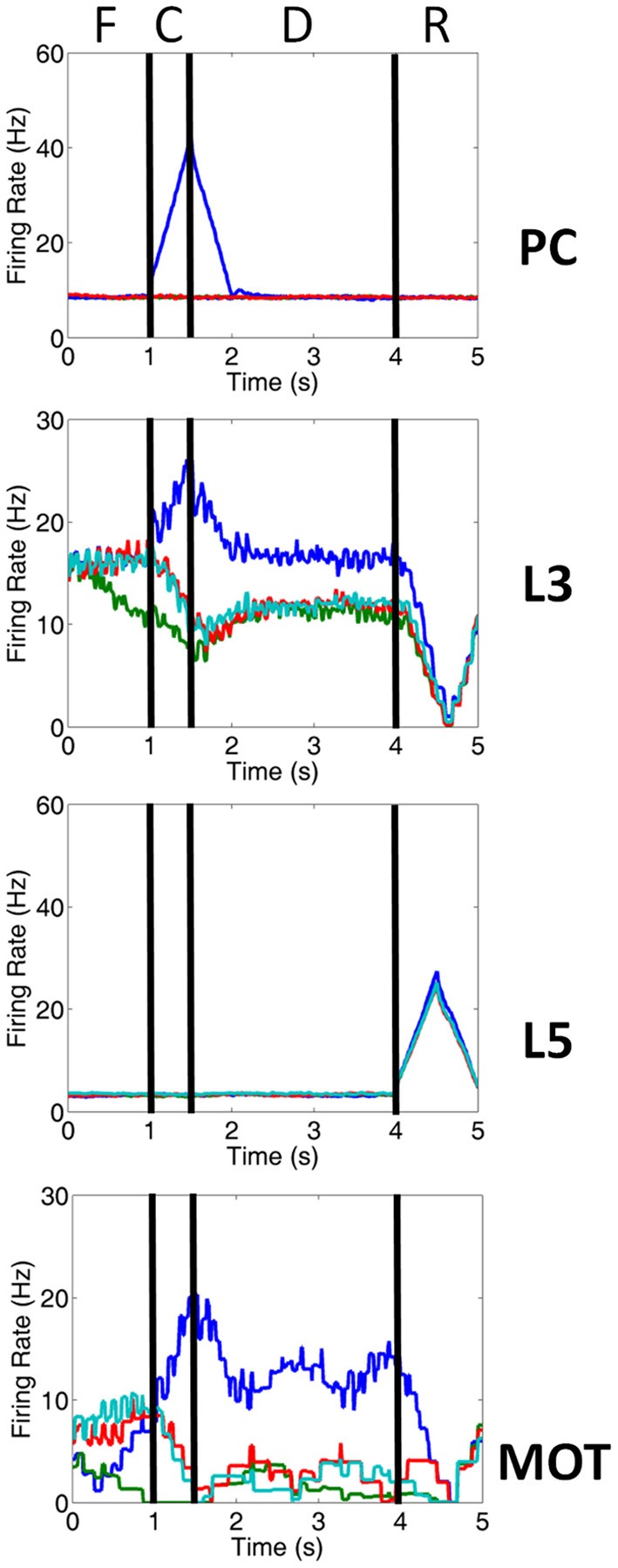
**Firing rate activity of neurons in the PC, PFC, and MOT for a single trial**. Typical firing rate activity of PC, layer 3, layer 5 and MOT neurons during a single working memory trial when DA and NE levels were optimal. PC neurons encoding the preferred direction (blue) are briefly activated when the cue is presented. Layer 3 neurons then hold onto this direction in working memory and drive neurons in the motor response layer, MOT. Layer 5 neurons, on the other hand, fire during the response phase of the task due to a corollary discharge mediated by the MD/SC and clear working memory in layer 3. Fixation (F), cue (C), delay (D) and response (R) periods are indicated at the top. Firing rates were smoothed using a simple moving average.

The dlPFC portion of the model contained four, two layer cortical columns representing visuospatial working memory circuits in the dlPFC, in which each column had a preferred saccade direction of 0, 90, 180, or 270° (Figure 1A). The two layers make up the deep supragranular (layer 3) and upper infragranular (layer 5) layers. Our current understanding of the microcircuity of the dlPFC suggests that supragranular is where working memory activity occurs and the infragranular layers is where response-related activity is located (Arnsten et al., 2012). The supragranular layers of each of the four columns receive visual input from four different parietal cortex (PC 7a) layers and from lateral excitatory and inhibitory connections within the PFC as shown in Figure 1A (Goldman-Rakic, 1995). These neurons fire in response to the stimulus, hold delay related activity in working memory, and are modulated by D1, α2A, and α1 receptors (Figures 2A, 3). Each supragranular layer in a column is also involved in biasing motor outputs through projections to four motor output (MOT) areas, which accumulate evidence in order to make a saccade direction decision (Schall et al., 2011). Between areas in MOT there is lateral inhibition, promoting competition.

Figures 1A, 2A show that infragranular layers receive subcortical inputs from the superior colliculus via the mediodorsal thalamus (MD/SC layer) (Stepniewska and Kosmal, 1986; Sommer and Wurtz, 2006). The SC→ MD→ PFC pathway has been studied in detail and it has been suggested that the response in infragranular layers is from a corollary discharge that takes place after an eye movement and acts as an efference copy of the motor movement (Wang et al., 2004; Sommer and Wurtz, 2008). The corollary discharge was an external input in our model that was simulated by briefly driving MD/SC neurons with Poissonian spike trains (40 Hz) for 500 ms at the beginning of the response phase (4 s into the trial). MD/SC neurons drove infragranular (layer 5) neurons in all cortical columns as can be seen in the L5 firing rate plot in Figure 3. L5 neurons, in turn, output to the BG layer (Arnsten, 2011), which sends inhibitory projections to all cortical columns, clearing working memory in infragranular layers. The clearing of working memory mediated by BG inhibitory projections to L3 can be seen in the dip in firing rates of L3 neurons during the response phase in Figure 3. This “cortico-striatal loop,” which acts as a means for updating working memory, has been suggested by Frank and colleagues (Frank et al., 2001; Frank, 2011). In their model, however, they use the basal ganglia as a means for selectively gating information *into* working memory, rather than clearing information from working memory as we suggest.

To construct our model, we used a publicly available simulator, which has been shown to simulate large-scale spiking neural networks efficiently and flexibly (Richert et al., 2011). The model contained a total of 57,212 neurons and approximately 30 million synapses. The number of neurons in each area is shown in Table 1. Connection probabilities in our cortical column model, which were adapted from Wagatsuma et al. (2011), can be found in Table 2. Within each column layer, there are excitatory–excitatory, excitatory–inhibitory, inhibitory–excitatory, and inhibitory–inhibitory connections (not shown in Figure 2A). There are no connections existing between layer 3 and layer 5 neurons within a column because the model aimed to match the response profiles of the groups to empirical data (see, e.g., Arnsten et al., 2012), rather than understand their interaction. All other connections probabilities between neural groups were set equal to 0.1. The connections that exist between groups are shown in Table 3. The simulation consisted of 50 trials at 6 s per trial for each set of parameters. Thus, the total simulation time was 5 min, which took approximately 37 min to run on a Tesla M2090 GPU.

**Table 1:** **Number of neurons in each area of the network**.

**Neural area**	**Excitatory neurons**	**Inhibitory neurons**
**SUBCORTICAL**		
BG	–	1000
MD/SC	1000	–
VTA (dopamine)	1000	–
LC (norepinephrine)	1000	–
**CORTICAL COLUMN**		
Layer 3	2585	729
Layer 5	606	133
**OTHER CORTICAL**		
PC 7a	1000	–
MOT	1000	–

**Table 2:** **Cortical connection probabilities within a column**.

**From**				
**To**	**L3e**	**L5e**	**L3i**	**L5i**
L3e	0.3584	0.0000	0.1552	0.0000
L5e	0.0000	0.0758	0.0000	0.3765
L3i	0.1008	0.0000	0.1371	0.0000
L5i	0.0000	0.0566	0.0000	0.3158

**Table 3:** **Cortical connection types between groups**.

**From**									
**To**	**PC7a(1–4)**	**L3e(1–4)**	**L3i(1–4)**	**L5e(1–4)**	**L5i(1–4)**	**MOTe(1–4)**	**MOTi(1–4)**	**MD/SC**	**BG**
PC7a(1–4)	–	–	–	–	–	–	–	–	–
L3e(1–4)	1-to-1	Full	1-to-1	–	–	–	–	–	–
L3i(1–4)	1-to-1	Full	–	–	–	–	–	–	–
L5e(1–4)	–	–	–	1-to-1	1-to-1	–	–	1-to-All	1-to-All
L5i(1–4)	–	–	–	1-to-1	1-to-1	–	–	–	
MOTe(1–4)	–	1-to-1	–	–	–	–	1-to-1	–	
MOTi(1–4)	–	–	–	–	–	Full	–	–	
MD/SC	–	–	–	–	–	–	–	–	–
BG	–	–	–	All-to-1	–	–	–	–	–

#### Neuron model

The Izhikevich model was used to govern the dynamics of the spiking neurons in this simulation. The computational efficiency of these point neurons (single compartment) makes them ideal for large-scale simulations. Izhikevich neurons are also highly realistic and are able to reproduce at least 20 different firing modes seen in the brain, which include: spiking, bursting, rebound spikes and bursts, sub threshold oscillations, resonance, spike frequency adaptation, spike threshold variability, and bistability of resting and spiking states (Izhikevich, 2004). Inhibitory and excitatory neurons in the cortex were modeled using the simple Izhikevich model, which are described by the following equations (Izhikevich, 2003):

(1) $$\dot{v}\;=0.04v^{2}+5v-u-I*\mu_{DA,\;NE,\text{ grp}}$$

(2) $$\dot{u}\;=a(bv-u)$$

(3) if v =30, then v=c,u=u+d

where *v* is the membrane potential, *u* is the recovery variable, *I* is the input current, μ is a neuromodulatory factor, and *a, b, c, d* are parameters chosen based on the neuron type. For regular spiking, excitatory neurons, we set *a* = 0.01, *b* = 0.2, *c* = −65.0, *d* = 8.0. For fast-spiking, inhibitory neurons, we set *a* = 0.1, *b* = 0.2, *c* = −65.0, *d* = 2.0. μ is a neuromodulatory factor that is dependent upon the dopamine concentration (*DA*), norepinephrine concentration (*NE*), and neural group grp. Neuromodulatory factors are summarized in Table 4 and explained in more detail in the Neuromodulation section below.

**Table 4:** **Neuromodulatory factor (μ) effects for differing neuromodulatory concentrations**.

	**Low DA + low NE**			**Low DA + optimal NE**			**Low DA + high NE**		
	**AMPA**	**NMDA**	***I***	**AMPA**	**NMDA**	***I***	**AMPA**	**NMDA**	***I***
L3 exc(pref) → L3 exc(pref)	0.1	10	–	0.1	15	–	0.1	15	–
L3 exc(npref) → L3 exc(pref)	1.4	1.4	–	1.4	1.4	–	1.4	1.4	–
L3 exc	–	–	–	–	–	–	–	–	0.8
	**Optimal DA + low NE**			**Optimal DA + optimal NE**			**Optimal DA + high NE**		
	**AMPA**	**NMDA**	***I***	**AMPA**	**NMDA**	***I***	**AMPA**	**NMDA**	***I***
L3 exc(pref) → L3 exc(pref)	0.1	10	–	0.1	15	–	0.1	15	–
L3 exc(npref) → L3 exc(pref)	1	1	–	1	1	–	1	1	–
L3 exc	–	–	–	–	–	–	–	–	0.8
	**High DA + low NE**			**High DA + optimal NE**			**High DA + high NE**		
	**AMPA**	**NMDA**	***I***	**AMPA**	**NMDA**	***I***	**AMPA**	**NMDA**	***I***
L3 exc(pref) → L3 exc(pref)	0.1	10	–	0.1	15	–	0.1	15	–
L3 exc(pref) → L3 exc(npref)	1	1	–	1	1	–	1	1	–
L3 exc	–	–	0.8	–	–	0.8	–	–	0.67

#### Synapse model

The synaptic input, *I*, driving each neuron was dictated by simulated AMPA, NMDA, GABA_A_ and GABA_B_ conductances (Izhikevich and Edelman, 2008; Richert et al., 2011). The conductance equations used are well established and have been described in Dayan and Abbott (2001); Izhikevich et al. (2004). The total synaptic input seen by each neuron was given by:

(4) $$\begin{array}{l} I=g_{\text{AMPA}}(v-0)+g_{\text{NMDA}}\frac{{\left[\frac{v+80}{60}\right]}^{2}}{1+{\left[\frac{v+80}{60}\right]}^{2}}(v-0)\\ \;+g_{{\text{GABA}}_{\text{A}}}(v+70)+g_{{\text{GABA}}_{\text{B}}}(v+90) \end{array}$$

where *v* is the membrane potential and *g* is the conductance. The conductances change according to the following first order equation:

(5) $${\dot{g}}_{i}=-\frac{g}{\tau_{i}}$$

where τ_*i*_ = 5, 100, 6, 150 ms for *i* = AMPA, NMDA, GABA_A_, GABA_B_ conductances, respectively. When an excitatory (inhibitory) neuron fires, gAMPA and gNMDA (gGABAA and gGABAB) increase by the synaptic weight, *w*μ_*i, DA, NE*.conn_, between pre- and post-synaptic neurons. μ, in this case, is a neurmodulatory factor that is dependent on the conductance (*i*), dopamine concentration (*DA*), norepinephrine concentration (*NE*), and connection (conn). Neuromodulatory factors are summarized in Table 4 and explained in more detail in the Neuromodulation section below. If not otherwise specified in Table 4, μ is set equal to 1.

### Neuromodulation

Our model incorporated simulated D1, α2A, and α1 receptors (Figure 2B). To understand the action of these receptors, it is first important to make clear the distinction between “preferred” and “non-preferred” directions and inputs. A neuron, for example, that shows persistent firing for a cue presented at 180° has a “preferred direction” of 180°. Preferred inputs to these neurons are excitatory inputs that also show persistent firing for a cue presented at 180° (i.e., recurrent excitatory connections, within a column). Non-preferred inputs are excitatory connections from neural groups that have other preferred directions, such as 0 or 90° (i.e., lateral excitatory connections, between columns). As discussed below, D1 receptors enhance non-preferred excitatory synapses onto preferred excitatory neurons (lateral excitation) and α2A receptors enhance excitatory connections for neurons encoding the same preferred direction (recurrent excitation).

D1 receptors have been shown to be important for blocking non-preferred excitatory inputs to cortical columns in the dlPFC (Arnsten, 2011). D1 receptors mediate the blocking of non-preferred inputs by increasing cAMP levels in spines where non-preferred inputs synapse onto preferred inputs (Vijayraghavan et al., 2007). Thus, when dopamine levels are low in PFC (weakly activating D1 receptors), non-preferred inputs to columns are enhanced. When dopamine levels are optimal, non-preferred inputs are weakened (see Figure 2B). At high levels of dopamine, which may occur during stress, it has been suggested (Arnsten, 2009) that cAMP levels in dendritic spines increase to the point that they weaken all inputs to dlPFC neurons (Figure 2C).

In contrast to D1 receptors, α2A receptors have been shown to be important for enhancing preferred excitatory inputs (i.e., inputs that code for the same saccade direction) within a cortical column in the dlPFC (Arnsten, 2011). Thus, when norepinephrine levels are low in PFC (weakly activating α2A receptors), recurrent excitatory inputs are weakened (see Figure 2B). When norepinephrine levels are optimal, on the other hand, recurrent excitatory inputs are enhanced within a column and an item can be held more robustly in working memory. This has been shown to occur by blocking cAMP in the dendrite (Wang et al., 2007). When NE levels are high, α1 receptors are activated due to a weaker affinity between NE and α1 receptors than NE and α2A receptors (Figures 2B,C). This causes a similar affect as highly stimulated D1 receptors and blocks all inputs (recurrent excitatory, lateral excitatory, and lateral inhibitory) to neurons (Mao et al., 1999).

We simulate the enhancement of non-preferred inputs when DA levels are low by increasing the strength of lateral excitatory connections (i.e., AMPA and NMDA conductances; see Equation 5) between columns encoding different preferred directions. When DA levels were low, then, μ in Equation 5 was set equal to 1.4 for AMPA and NMDA conductances on connections from non-preferred to preferred L3 excitatory connections (Table 4). When DA levels were optimal, μ was set equal to 1.0 for AMPA and NMDA conductances on connections from non-preferred to preferred L3 excitatory connections. When dopamine levels are high, it has been shown that, not only non-preferred inputs, but *all* inputs to dlPFC neurons are weakened as shown in Figure 2C (Vijayraghavan et al., 2007). This was simulated by setting μ equal to 0.8 in Equation 1, which decreases the overall input to a neuron. These neuromodulatory factors were chosen to match experimental data (Vijayraghavan et al., 2007), which suggest low overall activity and spatial tuning degradation in dlPFC with high DA levels and a high overall activity and spatial tuning degradation in dlPFC at low DA levels.

We simulate α2A receptor affects by decreasing the strength (i.e., NMDA conductances; see Equation 5, Table 4) of recurrent excitatory connections within a column when NE levels are low and increasing the strength of recurrent excitatory connections when NE levels are optimal and high (see Figure 2B). Thus, when NE levels were low, μ in Equation 5 was set equal to 10 for NMDA conductances on recurrent excitatory connections of L3 neurons within a column. When NE levels were optimal and high, μ was increased to 15 for NMDA conductances (see Table 4). In all of these cases, μ was set equal to 0.1 for AMPA conductances. A stronger influence of NMDA receptors on recurrent excitatory connections has been suggested previously as a means for increasing the stability of persistent states (Wang, 1999; Brunel and Wang, 2001). High NE levels, which activate α1 receptors, were simulated by multiplying the total synaptic current to a cell (see Equation 1) by a factor of 0.8. This factor was chosen to match experimental data seen in Birnbaum et al. (2004), where an α1 agonist was applied resulting in decreased firing rates and spatial tuning degradation in the dlPFC. When both NE and DA levels were high, we multiplied the total synaptic current to a cell by 0.67.

### Input presentation and saccade generation

The input to our network was structured according to the oculomotor delayed response (ODR) behavioral paradigm (Goldman-Rakic, 1995). Each individual experiment can be broken down into four stages: fixation, cue, delay, and response (Figure 1B). During the fixation stage, a constant, random Poissonian input drove all four columns in the network. The cue was presented for 500 ms at the 0° location, driving PC 7a inputs, and, hence, layer 3 neurons in the dlPFC encoding this saccadic direction (see blue line in PC chart of Figure 3). This biased drive was removed during the delay period, allowing for recurrent excitatory connections in a column to reverberate and hold onto the working memory. During the response period, Poissonian spike trains drove the MD/SC layer for 500 ms, simulating a corollary discharge, driving neurons in layer 5 of all columns. Layer 5 neurons, in turn, cleared working memory in layer 3 via GABAergic projections from the BG (Figure 3). The fact that response-related activity of layer 5 neurons is driven by a corollary discharge and PFC-BG interactions are involved in working memory updating is well established (Frank et al., 2001; Wang et al., 2004; Sommer and Wurtz, 2006). The behavioral response was obtained from the MOT layer, whose activity was biased by the layer 3 excitatory neurons in the dlPFC. To get the behavioral response, we chose the MOT group that had the greatest number of spikes in the 500 ms before the response period was cued. This is reminiscent of accumulator models that have been seen in decision making and proposed to exist in the brain in the MOT (Schall et al., 2011).

## Results

We first demonstrate in our results that we can match the inverted-U profiles seen with DA and NE manipulation. We then make predictions as to what working memory might look like in conditions that have not been explored experimentally, including: low DA + low NE, low DA + high NE, high DA + low NE, and high NE + high DA. Finally, we look at the behavioral responses of our model and show that they match well with those seen experimentally.

### Inverted-U DA and NE profiles

**Figure 4:**
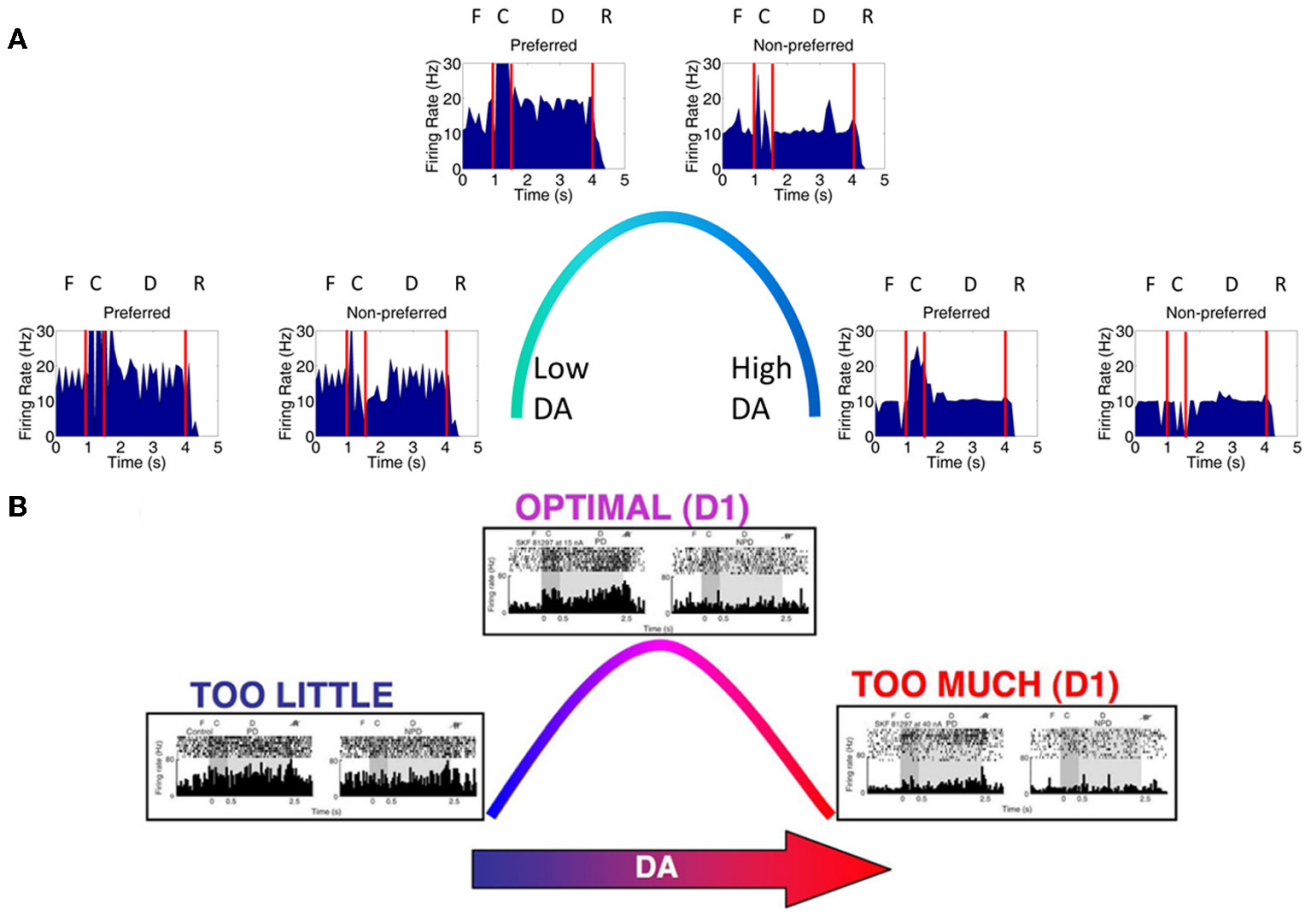
**Inverted-U dose-response with changing DA levels. (A)** Plot showing average firing rate summed over all neurons in layer 3 in a single trial. When DA levels were varied from low to high, we saw changes in the firing rate of working memory neurons that were consistent with those found experimentally. When DA levels were low, D1 receptors were only weakly activated causing an increase in the strength between columns (i.e., between non-preferred inputs). This lead to a degradation of spatial tuning as can be seen by both preferred (column encoding 0° in the model) and non-preferred (column encoding 90° in the model) columns showing high firing rates (left). The firing rates of preferred direction neurons vs. non-preferred direction neurons during the delay period were not significantly different (*p* > 0.1; *t*-test). When DA levels were high (right), all inputs to neurons in the PFC network were partially blocked due to D1 receptor over-stimulation, leading to a decrease firing rate to both preferred and non-preferred neurons (*p* > 0.1; *t*-test). When DA levels were optimal, preferred neuron firing rates were higher than non-preferred neurons as is characteristic in successful working memory traces (*p* < 10^−8^; *t*-test). **(B)** Experimental results obtained from Vijayraghavan et al. (2007); Arnsten (2011) showing a similar inverted-U with varying DA levels.

We first examined the responses of neurons in the four dlPFC columns of our model as we varied the concentration of DA and NE from low to high. The peristimulus spike histograms (PSTH) in Figure 4A shows average firing rate summed over all neurons in layer 3 during a single trial as DA levels vary from low to high. Figure 4A shows the changes in working memory responses for the preferred direction (0° column) and a non-preferred direction (90° column) when keeping the NE concentration optimal and varying the DA concentration (behavioral results found in Table 5). The columns coding for 180 and 270° had similar working memory patterns to the 90° column; however, we displayed the data in this way for ease of comparison with experimental data (Figure 4B). Our results show a similar inverted-U dose-response function as was seen in experimental results (Vijayraghavan et al., 2007) and shown in Figure 4B. That is, when DA concentrations are low (Figure 4A, left), both preferred and non-preferred columns show high firing rates due to excess noise (~20 Hz preferred, ~20 Hz non-preferred; *p* > 0.1 using a *t*-test comparing the firing rates of each column). This noise is caused by the strengthening of non-preferred inputs to all columns due to a weak activation of D1 receptors. When DA concentrations are high (Figure 4A, right), input to all neurons is diminished and the firing rates for both preferred and non-preferred directions decrease and the neurons lose their spatial tuning (~10 Hz preferred, ~10 Hz non-preferred; *p* > 0.1, *t*-test). This is thought to be caused by an increase in cAMP and may occur with high levels of stress (Arnsten, 2011).

**Table 5:** **Behavioral results—percentage of correct, incorrect, and no response behaviors**.

	**Low NE (%)**	**Optimal NE (%)**	**High NE (%)**
Low DA	70, 30, 0	68, 32, 0	20, 80, 0
Optimal DA	90, 10, 0	98, 2, 0	62, 38, 0
High DA	4, 0, 96	62, 38, 0	68, 0, 32

**Figure 5:**
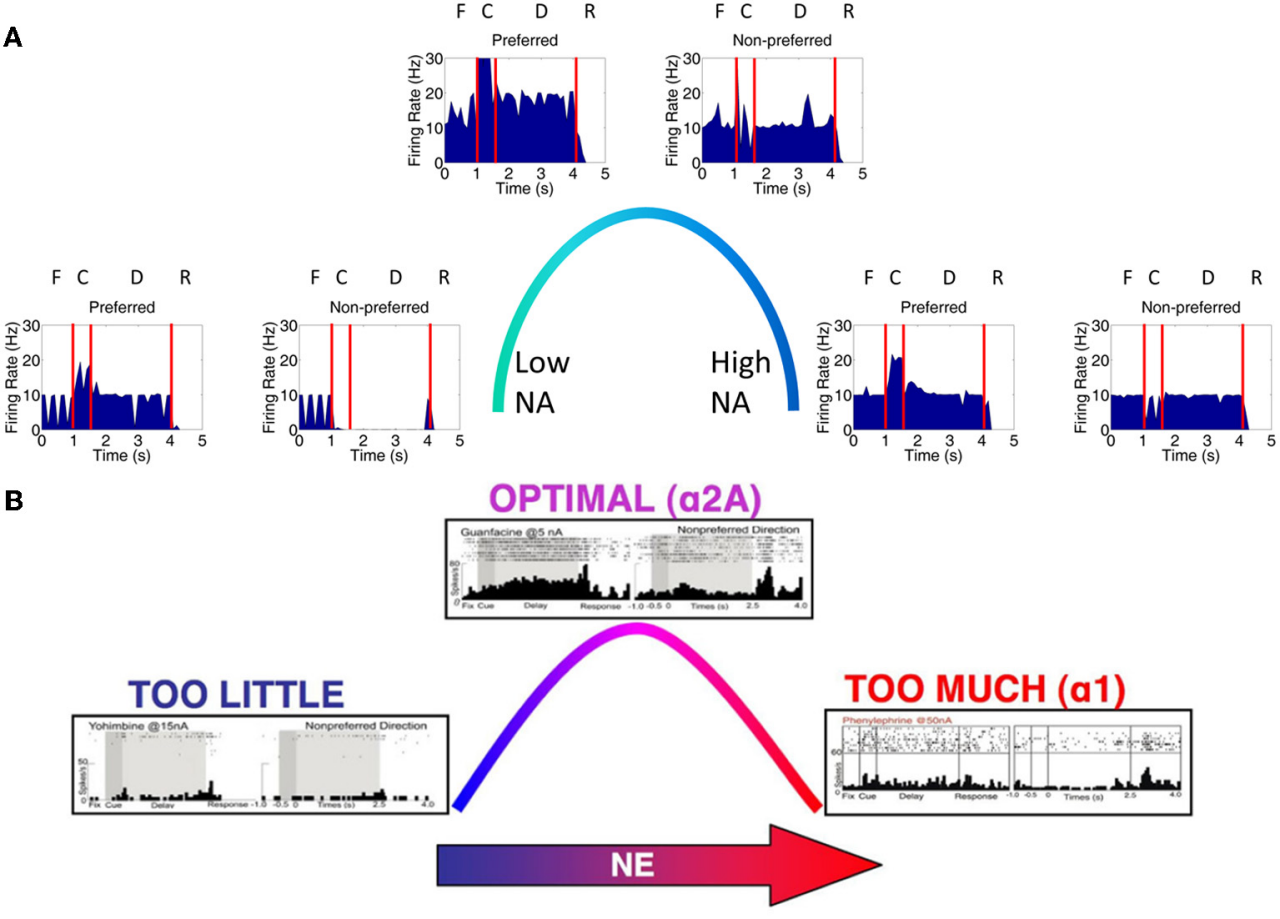
**Inverted-U dose-response with changing NE levels. (A)** When NE levels were varied from low to high, we saw changes in the firing rate of working memory neurons that were consistent with those found experimentally. When NE levels were low (left), α2A receptors were only weakly activated causing a decrease in the strength of recurrent connections within a column and, ultimately, a degradation of working memory as can be seen by the low firing rates in both preferred (column 1) and non-preferred (column 2) neurons. The firing rates of preferred direction neurons vs. non-preferred direction neurons during the delay period were significantly different since non-preferred direction neurons showed no response at all (*p* < 10^−8^; *t*-test). When NE levels were high (right), all inputs to neurons in the PFC network were partially blocked due to α1 receptor stimulation, leading to a decrease firing rate to both preferred and non-preferred neurons (*p* > 0.05; *t*-test). When NE levels were optimal, preferred neuron firing rates were higher than non-preferred neurons as is characteristic in successful working memory traces (*p* < 10^−8^; *t*-test). Note that the optimal and high NE conditions are the same as in Figure 4A due to the fact that identical neuromodulatory changes in the network are imposed in each of these states. **(B)** Experimental results from Birnbaum et al. (2004); Wang et al. (2007); Arnsten (2011) showing a similar inverted-U response with varying NE levels.

When keeping the DA concentration optimal and varying the NE concentration (Figure 5A), we also find a similar inverted-U dose-response function as was shown in Arnsten (2011). The PSTH in Figure 5A shows average firing rate summed over all neurons in layer 3 during a single trial as NE levels vary. This result matches well with the experimental result shown in Figure 5B. When NE concentrations are low (Figure 5A, left), recurrent excitatory connections within a column are weakened due to decreased activation of α2A receptors. This causes a decrease in the firing rates of both preferred and non-preferred columns and impairs delay-related firing. It has been shown that α2A activation may enhance firing by inhibiting cAMP (Wang et al., 2007). When NE concentrations are high (Figure 5A, right), inputs to all neurons are diminished (~10 Hz preferred, ~10 Hz non-preferred; *p* > 0.05, *t*-test) due to activation of α1 receptors and the firing rates for both preferred and non-preferred directions decrease (Mao et al., 1999; Birnbaum et al., 2004). These two receptors are activated at different concentrations of NE due to their different affinities for NE. That is, α2A receptors have a high affinity for NE and are activated at lower NE concentrations, while α1 receptors have a low affinity for NE and are activated at high NE concentrations.

When both NE and DA levels are optimal (Figures 4A, 5A, center), α2A and D1 receptors are optimally activated and the spatial tuning of the working memory columns are enhanced (~20 Hz preferred, 10 Hz non-preferred; *p* < 10^−8^, *t*-test). This is due to the fact that, in our model, when α2A receptors are optimally activated there is a strengthening of recurrent excitatory connections in layer 3 within each column. This, along with lateral inhibition, allows for the preferred column to be activated and stable at a persistently higher firing rate than non-preferred columns. When D1 is optimally active, excitatory connections between columns are weakened, decreasing the amount of noise that can activate any one column.

### Making predictions for combined high and low NE and DA levels

**Figure 6:**
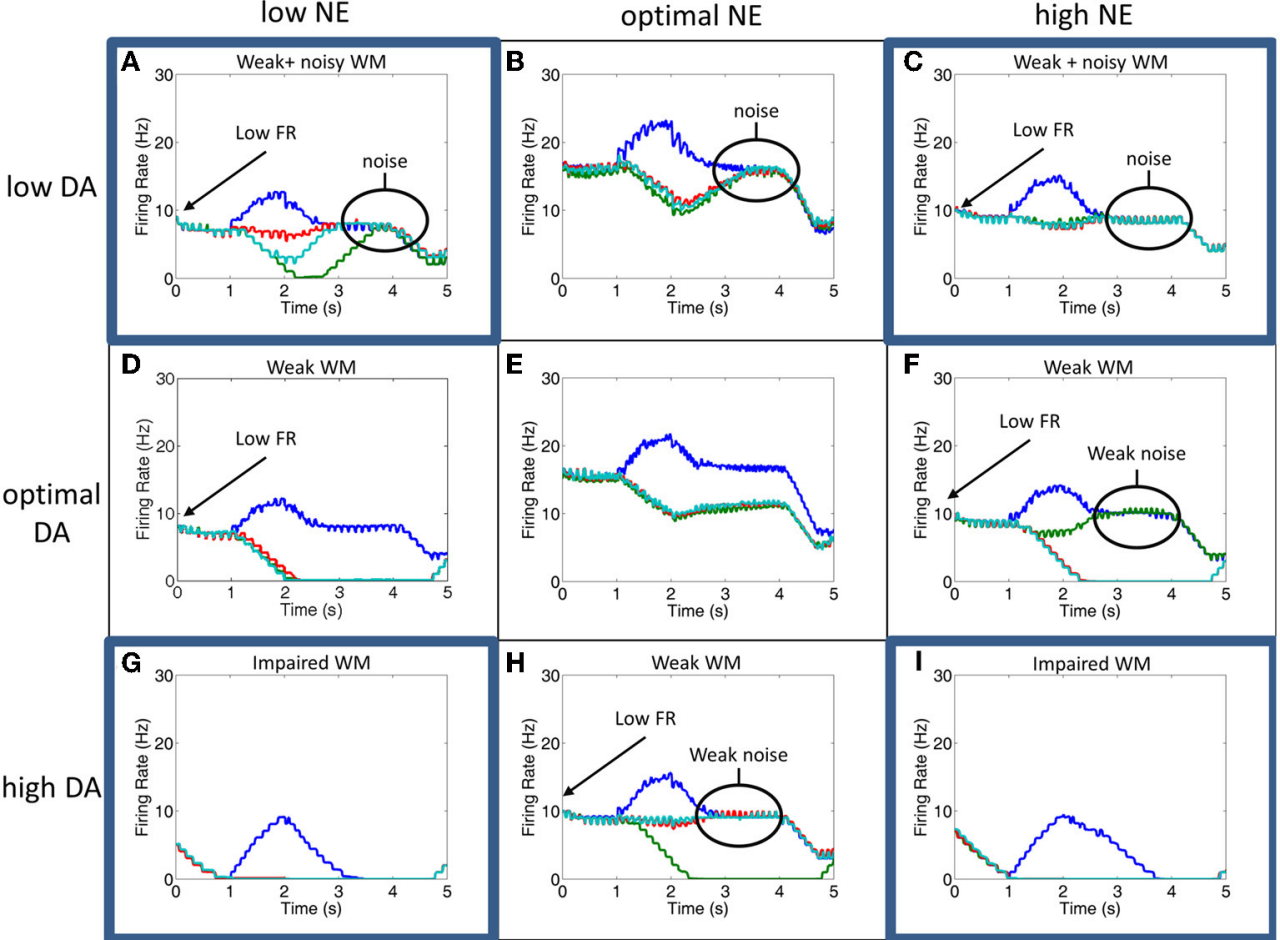
**Simultaneous alteration of NE and DA levels**. Figure shows firing rates for the all neurons of the four columns for low, optimal, and high concentrations of DA and NE. The column encoding the 0° saccade direction is shown in blue, 90° saccade direction in green, 180° in red and 270° in teal. Panels **(B,E,H)** and **(D–F)** are averages of the results seen in Figures 5, 6, respectively. The four corner conditions include: low DA + low NE **(A)**, low DA + high NE **(C)**, high DA + low NE **(G)**, and high NE + high DA **(I)**. To our knowledge, these four conditions have not been experimentally tested. Firing rates were smoothed using a simple moving average.

VTA and LC have reciprocal connections with each other and the PFC, suggesting that the activities of these two regions are highly dependent upon one another and that DA and NE concentrations should co-vary (Sara, 2009). Experimental studies, however, only manipulate one of these neuromodulators locally, potentially leaving the other at an optimal level. To understand how working memory changes when both NE and DA are at non-optimal levels, we also ran our model when both DA and NE were low, when DA and NE were high, when DA was high and NE was low, and when DA was low and NE was high. Figure 6 depicts what happens in these four cases (4 corners of the figure), as well in the optimal cases as has been shown in Figures 4, 5. We discuss the results of each of the four cases below.

Weak and noisy delay related activity results when both NE and DA levels are low (Figure 6A). When NE is low, excitatory recurrent activity is decreased due to decreased α2A receptor activation, leading to low firing rates. When DA is low, the strength of non-preferred synapses is increased due to decreased D1 receptor activation, leading to an increase in the amount of noise and a degradation of spatial tuning. In this case, the general pattern of the firing rates looks very similar to the case in which DA is low and NE is optimal, except with lower firing rates. This low firing rate may lead to an attentional deficit in subjects *in vivo* due to a weaker top-down signal.

We see a similar result when DA is low and NE is high (Figure 6C). In this case, high NE decreases the overall input to the neurons due to activation of α1 receptors, which has a similar effect on the overall firing rate as decreasing recurrent activity as seen in the low NE case. Because lateral inhibition is weakened in addition to recurrent excitation (see Figure 2C), however, a further degradation in spatial tuning may result with stimulation of alpha1 receptors that wasn't seen when NE levels were low and alpha2A receptors were only weakly stimulated. This weakening of lateral inhibition can be seen in comparing the low DA/high NE plot (Figure 6C) with Figures 6B,F. In Figures 6B,F, when the stimulus is presented the firing rate of all non-preferred directions simultaneously decrease as the preferred direction increases due to lateral inhibition. In Figure 6C, however, we see that, while some firing rates decrease with stimulus presentation (red line), others are only weakly affected (teal line). This is discussed further in the Behavioral Results section. As in the low DA and low NE case above, the low firing rate in this case may lead to an attentional deficit in subjects *in vivo* due to a weaker top-down signal. In addition to the low firing rates, the network is noisy due to decreased activation of D1 receptors as in the low DA and low NE case.

High DA combined with low NE (Figure 6G) led to significant impairments in working memory by causing very low overall firing rates. In this case, low NE decreases the strength of recurrent excitatory connections due to inactivation of α2A receptors. High DA decreases the overall input to the working memory neurons. As can be seen in Figure 6G, the neural groups are no longer able to persistently fire during the delay period, suggesting that it will have a weak or no influence on the saccade generation process in MOT since it depends on activity in the last 500 ms before the response period. This is explained in more detail in the Behavioral Results section.

A similar result is seen when both NE and DA levels are high (Figure 6I). This happens as a result of the activation of α1 receptors and the over-activation of D1 receptors, which both cause a decrease in the overall input to all working memory neurons in the columns. There is, however, a slightly longer tail of activation in the delay period than the high DA/low NE case, suggesting that performance will not be as bad behaviorally when both DA and NE are high as compared to the high DA/low NE case described above. Compared to the optimal NE/high DA and optimal DA/high NE cases, this state could potentially cause significant behavioral and attentional deficits depending on how strongly alpha1 and D1 receptors block inputs to PFC neurons. We chose neuromodulatory parameters such that some persistent activity would remain to drive behavioral responses, however, it is possible that high DA and high NE completely block inputs to PFC neurons, which would cause more severe impairments.

### Behavioral results

**Figure 7:**
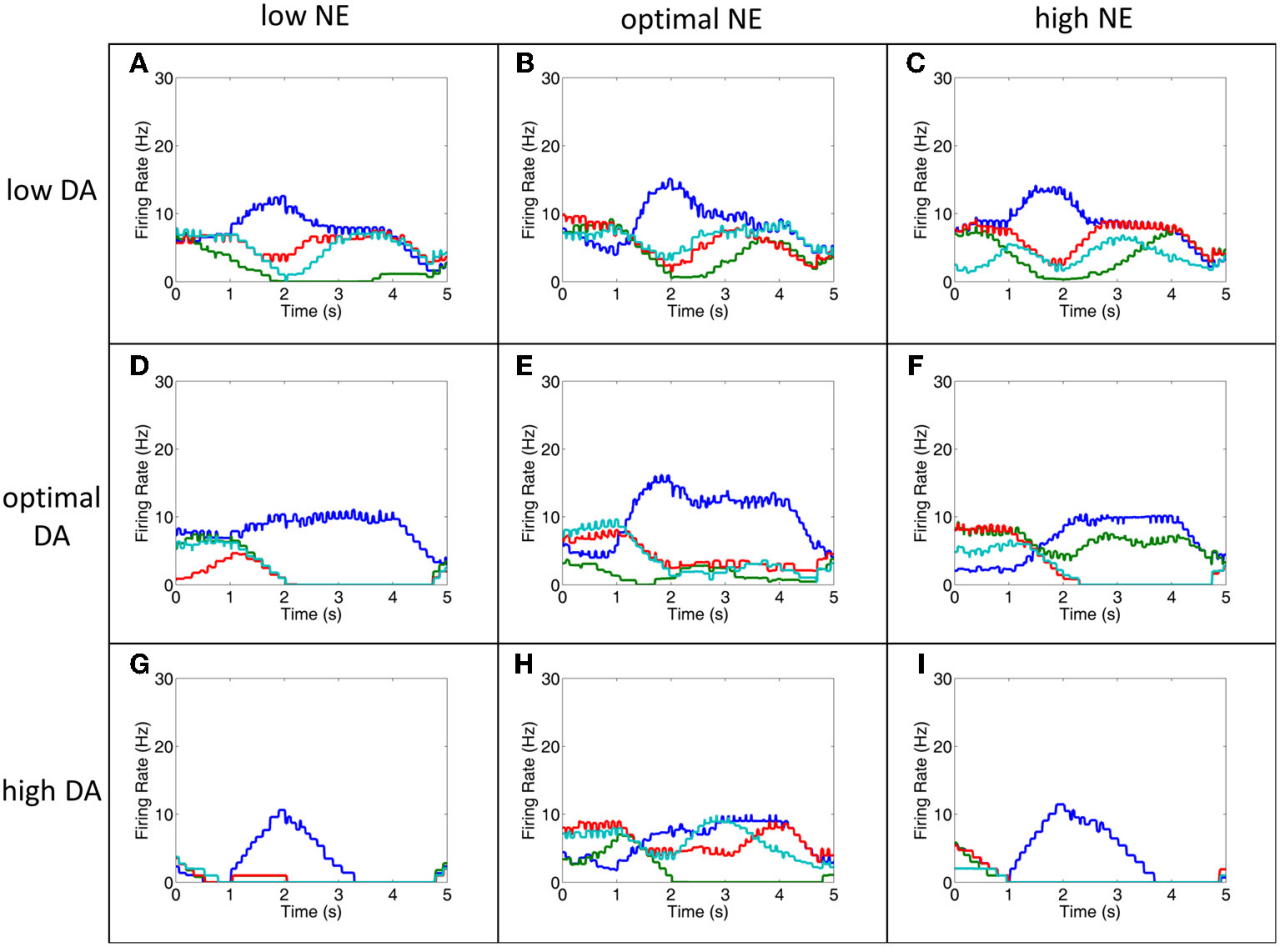
**The MOT filters out noise to improve behavioral performance**. This figure shows the firing rates of all MOT neurons for a single trial. During low DA **(A–C)**, optimal DA + high NE **(F)** and optimal NE + high DA **(H)** conditions, working memory in the PFC is extremely noisy and it is difficult to differentiate which of the columns would correctly drive the motor response (**D,E,G,I** do not show significant noise). As can be seen in this figure, however, some of this noise is filtered out in the MOT with lateral inhibition. Lateral inhibition allows the initially strong response from the preferred direction (blue, in these cases) to dominate and win out over other directions. This suggests that lateral inhibition may be a means for the MOT to improve behavioral performance even noise in the PFC is high. Firing rates were smoothed using a simple moving average.

We were able to capture how changes in neuromodulatory levels would affect behavior by having neurons in layer 3 project to MOT groups, which acted as a motor output layer. The responses of MOT neurons are shown in Figure 7 for low, optimal and high neuromodulatory level combinations. To get the behavioral response, we chose the MOT group that had the greatest number of spikes during the 500 ms prior to the response cue. This is reminiscent of accumulator models that have been seen in decision making and proposed to exist in the brain in the MOT (Schall et al., 2011). Table 5 shows the percentage of correct, incorrect, and null responses by the model. A null response occurs when no neuron fires during 500 ms before the response period.

Notice in comparing Figures 6, 7 that even when working memory is noisy due to low DA or high NE, behavioral performance may not significantly decline due to the architecture of the MOT. Specifically, because there is lateral inhibition in MOT, initially strong excitation of the preferred direction from L3 of the dlPFC will make it more likely it to “win out” over the other directions and choose the correct saccade direction. This is illustrated in comparing Figures 6, 7. Notice the low DA (Figures 7A–C), optimal DA + high NE (Figure 7F), and optimal NE + high DA (Figure 7H) conditions and compare them with the working memory plots in Figure 6. Working memory is extremely noisy during the last 3 s of the trial, however, since the earliest input to the MOT layer is most significant, lateral inhibition in the MOT will filter out the noise later in the delay period and the correct saccade direction will have a higher probability of being chosen. This suggests that lateral inhibition in the MOT may be another means for filtering out noise and optimizing behavioral performance.

Behavior was the worst when NE was low and DA was high as a result of extremely low firing rates in all layers (Figure 6G). This was due to weakened recurrent excitatory activity as a result of weakly activated α2A receptors and the overall input to the network was diminished due to overactive D1 receptors. As a result, the percentage of non-decision trials (meaning no neurons fired during last 500 ms before the response phase) was 96%. The only other case that had non-decision trials (32%) was when both NE and DA were high. Behavioral deficits were also quite high when NE was high and DA was low. This is due to activation of α1 receptors and inactivation of D1 receptors. Inactive D1 receptors strengthened non-preferred inputs and led to a large amount of noise leaking into each cortical column, whereas activation of α1 receptors decreased firing rates to all neurons. Low firing rates due to activation of α1 receptors (i.e., high NE) seems to hinder behavioral performance more than deactivation of α2A receptors (low NE), which also decrease firing rates. This behavioral deficit was explicitly due to noise as opposed to non-decision trials as discussed above in the low NE + high DA case.

Another interesting result found in our model is that behavior is significantly worse under optimal DA + high NE conditions compared to optimal DA + low NE conditions. In low NE conditions, α2A receptors are only weakly stimulated, which decreases recurrent activity, whereas, at high NE conditions, α1 receptors are activated and decrease all inputs to the neurons. Physiologically these changes have the same end result: a decrease in overall activity and a degradation of spatial tuning. Our behavioral results, however, further suggest that α1 activation may be more detrimental to behavior than α2A inactivation. Decreasing the overall input to a neuron (via α1 receptor activation) introduces more noise into working memory than decreasing recurrent excitatory activity (via α2A receptor deactivation). This is likely due to the fact that α1 receptor activation decreases recurrent activity *and* lateral inhibition between columns in dlPFC (see Figure 2C). Decreasing lateral inhibition degrades spatial tuning more so than decreasing recurrent excitation alone, leading to a greater amount of noise in the network. Thus, the strength of the signal, which depends on recurrent excitation, seems less important in terms of behavioral performance than the ability to block out noise with lateral inhibition. This highlights the importance of GABAergic neurons in working memory and behavioral performance.

Our behavioral results also match quite well with monkey behavioral results. Systemic injection of cirazoline, an α1 agonist, showed a 20% reduction in the number of correct responses (Birnbaum et al., 2004), whereas local application of yohimbine, an α2 antagonist, showed a 10% reduction in the number of correct responses (Li and Mei, 1994). These results match well with the above result suggesting that the optimal DA, high NE condition, which activates α1 receptors, is more detrimental to behavioral performance than the optimal DA, low NE condition, which weakens α2A receptor activation. Likewise, local application of a D1 antagonist caused an approximately 30% reduction in the number of correct responses (relate to our optimal NE, low DA case) and local application of a D1 agonist caused an approximately 20% reduction in the number of correct responses (relate to our optimal NE, high DA case).

## Discussion

The model presented in this paper is able to correctly match experimental data showing an inverted-U dose-response with varying levels of dopamine and norepinephrine. We showed that when DA was low, inactivation of D1 receptors strengthened non-preferred inputs to working memory columns, increasing the amount of noise in working memory. When NE was low, working memory is weak due to weak activation of α2A receptors on recurrent excitatory synapses. When either NE or DA levels were high, all inputs to the network are weakened due to over activation of D1 receptors and activation of α1 receptors. In addition, we make predictions regarding the type of working memory and behavioral deficits that might occur in conditions that have not been experimentally tested. For example, when DA levels were low and NE levels were high, we saw significant behavioral deficits resulting from a large amount of noise in working memory. When DA levels were high and NE levels were low, on the other hand, we saw behavioral deficits due to weak overall network activity. We were also able to show that lateral inhibition in working memory may play a more important role in increasing signal-to-noise ratio than increasing recurrent excitatory input. Lateral inhibition in MOT also plays an important role in reducing noise in the motor output in order to improve behavioral performance when working memory was noisy.

The importance of neuromodulators in working memory has been known for decades (Brozoski et al., 1979). Today, we not only know the specific role that dopaminergic and noradrenergic receptors play in working memory, we also know intracellular synaptic signaling events that likely occur as a result of activation of these receptors (Arnsten et al., 2012). Due to the inherent complexity that arises with multiple interacting cortical circuits, it is important to test the experiment-based theories of how these circuits behave with models. We developed our model to test at the circuit level the current understanding of how noradrenergic and dopaminergic receptors modulate working memory and, further, come up with experimentally testable predictions.

Because of interactions between the LC and VTA (Sara, 2009), NE and DA levels may covary in dlPFC. Thus, an important prediction of our model is how working memory and behavior might change when both NE and DA are low or high. When both NE and DA levels are low (Figure 6A), we observe that working memory in the model is both noisy and dlPFC neurons have a low firing rate due to weak activation of D1 and alpha2A receptors, respectively. Behavioral results, however, in this condition were comparable to the low DA/optimal NE condition, suggesting that weakening recurrent excitation alone does not have a profound effect on behavior.

When DA levels are low and NE levels are high (Figure 6C), working memory is also noisy and has a low firing rate due to weak activation of D1 receptors and activation of alpha1 receptors, respectively. This led to a significant behavioral deficit. That is, it is *worse* behaviorally to have a low firing rate due to activation of α1 receptors (high NE) than deactivation of α2A receptors (low NE). This can also be seen in comparing the optimalDA + lowNE (Figure 6D) and the optimalDA + highNE (Figure 6E) cases. This suggests that it is more optimal to decrease the overall recurrent excitation than decrease the overall input to excitatory neurons. This is likely due to the fact that activation of α1 receptors decreases both recurrent excitation *and* lateral inhibition, which degrades spatial tuning and introduces noise into the network. This further highlights the importance of GABAergic neurons for working memory and points to the fact that it may be more important for working memory and behavioral performance to block noise with strong lateral inhibition than increase the “signal” with strong excitation.

When both NE and DA levels are high (Figure 6I), we observe that our working memory is significantly impaired due to an overall low activity in the PFC. A similar result is seen when DA is high and NE is low (Figure 6G). Behavioral deficits, however, are much worse in the highDA + lowNE case. That is, it is *better* behaviorally to have a low firing rate due to activation of α1 receptors (high NE) than deactivation of α2A receptors (low NE) in a high DA situation. This is interesting because it contradicts the low DA case explained above, suggesting an important role that noise might play in increasing behavioral performance when NE levels are low.

These results are especially interesting because PFC projects to both the VTA and LC and the VTA and LC have reciprocal projections to each other and respond to similar stimuli (Sara, 2009). Therefore, it is quite likely that the concentrations of these two neuromodulators are dependent upon one another. For example, high DA levels may excite neurons that project to the LC, leading to high NE levels and vice versa. It may also be that changes in DA levels counteract changes in NE levels in order to optimize behavior via reciprocal connections to the VTA and LC. This could be tested experimentally by manipulating DA levels and measuring NE levels or stimulating the VTA and LC and measuring the levels of NE and DA, respectively. Experimental procedures typically involve manipulating only of these neuromodulators and seeing how this affects working memory. Simultaneously changing DA and NE concentrations, however, seems more biologically plausible and is easily implemented experimentally. It will be interesting to test this in the future to verify or disprove our model.

Our model also suggests that the corollary discharge from the SC projects to layer 5 neurons in the PFC and that this signal is important for clearing working memory. The idea that corollary discharge projects to layer 5 neurons is not new (Wang et al., 2004). Layer 5 neurons in the PFC show firing timed with the saccadic response during an ODR task. Wang et al. showed that this response is attenuated by applying a D2 antagonist. Because D2 receptors have been shown to be important for cognitive flexibility (Floresco et al., 2006) and layer 5 neurons project to the basal ganglia, which has also been shown to be important for updating working memory (Frank et al., 2001), we proposed this SC→PFC→BG circuit for clearing working memory. It will be interesting to verify this circuitry and investigate the D2 receptor's role in working memory updating in addition to the computational role that the corollary discharge plays in informing the rest of the brain that a movement has just been made (Sommer and Wurtz, 2008).

Several interesting models of working memory have been recently developed (Compte et al., 2000; Brunel and Wang, 2001; Mongillo et al., 2008; Szatmary and Izhikevich, 2010; Martinet et al., 2011; Chen et al., 2013; Hansel and Mato, 2013). Instead of relying solely on NMDA/AMPA ratios, Mongillo et al. (2008) and Hansel and Mato (2013) developed a working memory model that also uses short-term plasticity to drive persistence. Hansel and Mato found that short-term plasticity is particularly important for maintaining working memory when increasing the size of the network as well as introducing high variability in spiking as has been observed *in vivo*. Though we did not see any size-related deficits in working memory, it would be interesting to see in future studies whether our model is robust to such changes. Compte et al. (2000) developed a prefrontal cortical network model that demonstrated the importance of NMDA and GABA currents in maintaining a stable working memory and realized an excitatory–inhibitory circuit architecture that was necessary for iso-directional tuning. Brunel and Wang (2001) had several important findings, including showing the how external drive can affect the inverted-U shape, how distracting inputs can affect the network, and the importance of GABA and NMDA currents. These models were mainly focused on constructing a working recurrent neural model of working memory and trying to better understand mechanisms that might influence the stability of representations. Our model, on the other hand, goes a step deeper by focusing on recent experimental findings involving the location and effects that D1, α2A, and α1 receptors have on working memory networks. In this sense, our model builds upon these models by adding another layer of experimental detail that wasn't known when these models were developed.

A couple of important differences should be discussed in comparing our model with other models of working memory. First, many models of working memory include a large number of saccade directions so that these directions may be stored continuously as a “bump attractor” (Compte et al., 2000; Wei et al., 2012). Our model, on the other hand, only includes 4 different directions so we are not able to assess how neuromodulation might affect the stability and size of the attractor states. We speculate that decreasing NE would cause the attractor to become smaller or perhaps disappear, whereas decreasing DA would cause the attractor activity to spread, introducing uncertainty into the working memory representation. It would be interesting to see in the future how neuromodulator concentrations affect the spread and stability of these attractor states. Our model also has a unique way of choosing the saccade direction by incorporating a motor output layer with lateral inhibition. Other models (see, e.g., Compte et al., 2000) choose the saccade direction by decoding the memory trace using population vector decoding in the last several hundred milliseconds of the delay period. This would result in far poorer behavioral performance in our model due to the fact that recurrent inhibition in the motor output layer makes the decision heavily weighted on the initial excitation of the dlPFC neurons. Further modeling and experimental studies will be needed to assess the validity of either of these approaches.

In sum, this study focused on modeling the effects that D1, α2A, and α1 receptors have working memory. We were able to reproduce experimental results showing inverted-U dose-dependent changes that occur for differing dopamine and noradrenaline concentrations. In particular, we showed that D1 receptors were important for improving spatial tuning in working memory by blocking noise induced by lateral excitation in the PFC. α2A receptors, on the other hand, strengthened recurrent excitatory connections within a column, leading to more robust working memory representations. In addition, our model was able to predict how working memory and behavior will change under low DA + low NE, low DA + high NE, high DA + low NE, and high NE + high DA conditions, which has not yet been shown experimentally. Finally, we demonstrated the important role that inhibition plays in reducing noise in both working memory and motor output layers. We hope these results will solidify current experimental theories on working memory as well as provide researchers with testable predictions for non-standard experimental conventions that involve manipulating DA and NE levels simultaneously.

### Conflict of interest statement

The authors declare that the research was conducted in the absence of any commercial or financial relationships that could be construed as a potential conflict of interest.
